# Apoptosis induction of U937 human leukemia cells by diallyl trisulfide induces through generation of reactive oxygen species

**DOI:** 10.1186/1423-0127-19-50

**Published:** 2012-05-11

**Authors:** Yung Hyun Choi, Hyun Soo Park

**Affiliations:** 1 Department of Biochemistry, Dongeui University College of Oriental Medicine, San 45, Yangjung-dong Busanjin-gu, Busan, 614-052, Republic of Korea; 2 Department of Biomaterial Control and Anti-Aging Research Center & Blue-Bio Industry RIC, Dongeui University, 995 Eomgwangno Busanjin-gu, Busan, 614-714, Republic of Korea

**Keywords:** U937, DATS, Apoptosis, ROS, Caspase

## Abstract

**Background:**

Diallyl trisulfide (DATS) is one of the major constituents in garlic oil and has demonstrated various pharmacological activities, including antimicrobial, antihyperlipidemic, antithrombotic, and anticancer effects. However, the mechanisms of antiproliferative activity in leukemia cells are not fully understood. In this study, the apoptotic effects of DATS were investigated in human leukemia cells.

**Results:**

Results of this study indicated that treatment with DATS resulted in significantly inhibited leukemia cell growth in a concentration- and time-dependent manner by induction of apoptosis. In U937 cells, DATS-induced apoptosis was correlated with down-regulation of Bcl-2, XIAP, and cIAP-1 protein levels, cleavage of Bid proteins, activation of caspases, and collapse of mitochondrial membrane potential. The data further demonstrated that DATS increased intracellular reactive oxygen species (ROS) generation, which was attenuated by pretreatment with antioxidant *N*-acetyl-l-cysteine (NAC), a scavenger of ROS. In addition, administration of NAC resulted in significant inhibition of DATS-induced apoptosis by inhibiting activation of caspases.

**Conclusions:**

The present study reveals that the cytotoxicity caused by DATS is mediated by generation of ROS and subsequent activation of the ROS-dependent caspase pathway in U937 leukemia cells.

## Background

Reactive oxygen species (ROS), such as hydrogen peroxides (H_2_O_2_), hydroxyl radicals (OH·), and superoxide anions (·O_2_^-^) are commonly generated as a consequence of aerobic metabolism in mitochondria [1,2]. In normal physiological states, ROS are produced through electron leakage when molecular oxygen is used for ATP production in the mitochondrial electron transport chain [3-5]. As well as ROS production by mitochondria, these are also generated by various exogenous sources, such as chemicals and radiation [6-8]. Excessive accumulation of ROS results in severely demolished cellular macromolecules, such as DNA, and induces G2/M phase arrest by DNA damage [9-11]. Increased intracellular ROS also triggers apoptosis by activation of the intrinsic apoptotic pathway through induction of mitochondrial permeability transition and release of cytochrome *c* from mitochondria to the cytosol [4,12-14].

Garlic (*Allium sativum*) is a common plant used mainly as food and has recently been reported to have medicinal properties [15-17]. Many researchers have recently demonstrated that sulfur-containing compounds, such as diallyl sulfide (DAS), diallyl disulfide (DADS), and diallyl trisulfide (DATS), which are major components of garlic, may be associated with reduced risk of certain cancers [18,19]. These compounds are known to inhibit cell proliferation and increase apoptosis in various cancer cell lines [20-22]. In particular, DATS containing three sulfur atoms against DAS and DADS have been known to have stronger biological activity, such as anti-cancer and anti-inflammatory effects [23-26]. Recently, Das et al. [27] have shown that DATS induces apoptosis through activation of ROS-dependent caspase activation in human glioblastoma cells. Others studies have also supported the notion that DATS induces dramatic ROS generation in cancer cells through a mitochondrial pathway [26,28,29]. However, the cellular and molecular mechanisms underlying the compound have yet not been completely elucidated.

In the present study, we hypothesized that DATS would also induce functional changes in mitochondria in association with ROS generation in the course of apoptosis induction in human leukemia cells. To test this hypothesis, we evaluated the effects of DATS on mitochondrial membrane potential (MMP) values, ROS generation, and apoptosis using the human leukemia U937 cell line. Our results indicated the requirement of ROS generation in apoptosis induced by DATS in U937 cells.

## Methods

### Reagents and antibodies

DATS was purchased from LKT Laboratories (St Paul, MN). 3-(4,5-dimetylthiazol-2-yl)-2,5-diphenyl-tetrazolium (MTT), propidium iodide (PI), 5,5′, 6,6′-tetrachloro-1,1′,3,3′-tetraethyl-imidacarbocyanine iodide (JC-1), 4,6-Diamidino-2-phenyllindile (DAPI), 5-(and 6)-carboxy-2’7’-dichlorodihydrofluorescein diacetate (DCFDA), and *N*-acetyl-l-cysteine (NAC) were purchased from Sigma-Aldrich (St. Louis, MO). Fetal bovine serum (FBS) and caspase activity assay kits were obtained from GIBCO-BRL (Gaithersburg, MD) and R&D Systems (Minneapolis, MN), respectively. DNA staining kit (CycleTEST™ PLUS Kit) and enhanced chemiluminescence (ECL) kit were purchased from Becton Dickinson (San Jose, CA) and Amersham (Arlington Heights, IL), respectively. All antibodies were purchased from Santa Cruz Biotechnology (Santa Cruz, CA).

### Cell culture and MTT assay

The human leukemia cell lines used in our studies included human monocytic leukemia (U937 and THP-1), human acute myeloblastic leukemia (HL60) and human erythroid chronic myeloid leukemia (K562) were purchased from the American Type Culture Collection (Rockville, MD). They were maintained at 37°C in humidified 95 % air and 5 % CO_2_ in RPMI1640 supplemented with 10 % heat-inactivated FBS, 2 mM glutamine, 100 U/ml penicillin, and 100 μg/ml streptomycin. DATS was dissolved in dimethyl sulfoxide (DMSO) as a stock solution at a 100 mM concentration, and the stock solution was then diluted with the medium to the desired concentration prior to use. For the cell viability study, cells were grown to 70 % confluence and treated with DATS. Control cells were supplemented with complete media containing 0.1 % DMSO (vehicle control). Following treatment, cell viability was determined by use of the MTT assay, which is based on the conversion of MTT to MTT-formazan by mitochondrial enzymes [30]. The effect of DATS on inhibition of cell growth was assessed as the percentage of cell viability, where vehicle-treated cells were considered 100 % viable.

### Nuclear staining with DAPI

For DAPI staining, cells were washed with phosphate-buffered saline (PBS) and fixed with 3.7 % paraformaldehyde (Sigma-Aldrich) in PBS for 10 min at room temperature. Fixed cells were washed with PBS and stained with 2.5 μg/ml DAPI solution for 10 min at room temperature. Cells were then washed twice with PBS and analyzed using a fluorescence microscope (Carl Zeiss, Germany).

### DNA fragmentation assay

Following DATS treatment, cells were lysed in a buffer containing 10 mM Tris–HCl, pH 7.4, 150 mM NaCl, 5 mM EDTA, and 0.5 % Triton X-100 for 1 h at room temperature. Lysates were vortexed and cleared by centrifugation at 19,000 g for 30 min at 4°C. A 25:24:1 (v/v/v) equal volume of neutral phenol : chloroform : isoamyl alcohol (Sigma-Aldrich) was used for extraction of DNA in the supernatant, followed by electrophoretic analysis on 1.0 % agarose gels containing 0.1 μg/ml ethidium bromide (EtBr, Sigma-Aldrich) [31].

### Measurement of cell cycle, mitochondrial membrane potential (MMP) values and ROS generation by a flow cytometer

For analysis of the cell cycle, cells were collected, washed with cold PBS, and fixed in 75 % ethanol at 4°C or 30 min. A DNA staining kit was used according to the manufacturer’s instructions for measurement of the DNA content of the cells [32]. Flow cytometric analyses were carried out using a flow cytometer and the relative DNA content was determined using CellQuest software based on the presence of red fluorescence. MMP (*ΔΨm*) was determined using the dual-emission potential-sensitive probe, JC-1. Cells were collected and incubated with 10 μM JC-1 for 20 min at 37°C in the dark. Cells were then washed once with PBS and analyzed using a flow cytometer [33]. For measurement of ROS generation, cells were treated with DATS for various periods and the medium was discarded; cells were then incubated with new culture medium containing 10 μM of DCFDA at 37°C in the dark for 20 min. Cell lysates were used for evaluation of ROS generation using a flow cytometer [34].

### Protein extraction and Western blotting

Cells were harvested and washed twice in PBS at 4°C. Total cells lysates were lysed in lysis buffer (40 mM Tris (pH 8.0), 120 mM, NaCl, 0.5 % NP-40, 0.1 mM sodium orthovanadate, 2 μg/ml aprotinin, 2 μg/ml leupeptin, and 100 μg/ml phenymethylsulfonyl fluoride). Supernatants were collected and protein concentrations were then measured using protein assay reagents (Pierce, Rockford, IL). Equal amounts of protein extracts were denatured by boiling at 95°C for 5 min in sample buffer (0.5 M Tris–HCl, pH 6.8, 4 % SDS, 20 % glycerol, 0.1 % bromophenol blue, 10 % β-mercaptoethanol) at a ratio of 1:1, subjected to SDS-polyacrylamide gels, and transferred to polyvinylidene difluoride membranes (Schleicher & Schuell, Keene, NH) by electroblotting. Membranes were blocked with 5 % non-fat dry milk in PBS with Tween 20 buffer (PBS-T) (20 mM Tris, 100 mM NaCl, pH 7.5, and 0.1 % Tween 20) for 1 h at room temperature. Membranes were then incubated overnight at 4°C with the primary antibodies, probed with enzyme-linked secondary antibodies, and visualized using an ECL kit, according to the manufacturer's instructions.

### Caspase activity assay

Activities of caspases were determined by use of colorimetric assay kits, which utilize synthetic tetrapeptides (Asp-Glu-Val-Asp (DEAD) for caspase-3; Ile-Glu-Thr-Asp (IETD) for caspase-8; Leu-Glu-His-Asp (LEHD) for caspase-9, respectively) labeled with p-nitroaniline (pNA). Briefly, DATS-treated and untreated cells were lysed in the supplied lysis buffer. Supernatants were collected and incubated with the supplied reaction buffer containing DTT and DEAD-pNA, IETD-pNA, or LEHD-pNA as substrates at 37°C. The reactions were measured by changes in absorbance at 405 nm using the VERSAmax tunable microplate reader.

### Statistical analysis

Unless otherwise indicated, each result is expressed as the mean ± SD of data obtained from triplicate experiments. Statistical analysis was performed using a paired Student t-test. Differences at *p* < 0.05 were considered statistically significant.

## Results

### Inhibition of cell viability by DATS in human leukemia cells

**Figure 1:**
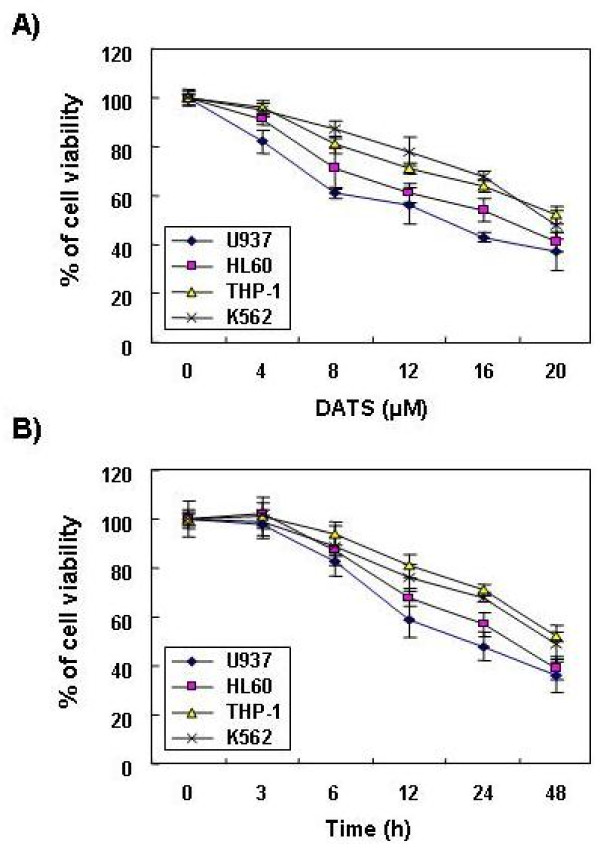
**Inhibition of cell viability by DATS treatment in human leukemia cells.** Cells (U937, THP-1, HL60 and K562) were plated at a concentration of 2.5 x 10^5^ cells per 60-mm plate. Following 24 h of stabilization, cells were treated with various concentrations of DATS for 48 h **(A)** or incubated with 20 μM of DATS for the indicated times **(B)**. Cell viability was determined by MTT assay. Results are expressed as percentage of the vehicle treated control ± SD of three separate experiments. A Student’s t-test (*, *p* < 0.05 *vs*. untreated control) was used for determination of significance.

**Figure 2:**
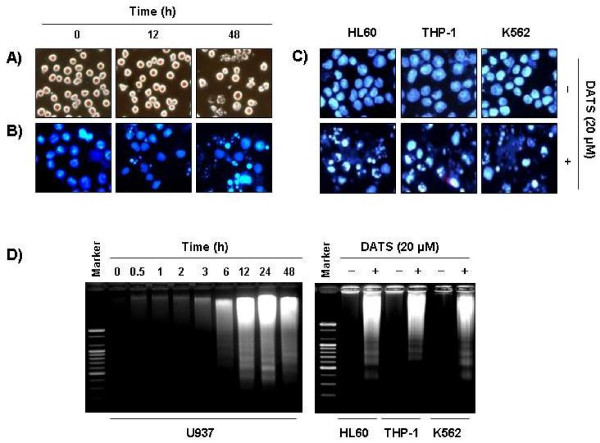
**Induction of apoptosis by DATS treatment in human leukemia cells.(A)** Following treatment with 20 μM of DATS for 12 and 48 h, U937 cell morphology was visualized using an inverted microscope. Magnification, ×200. **(B and C)** Cells grown under the same conditions as (A, U937) or cells (THP-1, HL60 and K562) treated with 20 μM of DATS for 48 h were fixed, stained with DAPI, and the nuclear morphology was then photographed under fluorescence using a blue filter. Magnification, ×400. **(D)** For analysis of DNA fragmentation, genomic DNA was extracted and then electrophoresed on a 1.0 % agarose gel, and then visualized by EtBr staining. Marker indicates a size marker of the DNA ladder.

To evaluate the effects of DATS on leukemia cell viability, four leukemia cell lines (U937, THP-1, HL60 and K562) were stimulated with various concentrations of DATS for 48 h or with 20 μM DATS for the indicated times, and an MTT assay was performed. As shown in Figure 1, DATS induced a decrease in cell viability in a dose- and time-dependent manner in all four models of leukemia. For example, treatment with 20 μM DATS for 24 h and 48 h in U937 cells resulted in 53 % and 62 % inhibition, respectively, which was associated with many morphological changes (Figure 2A).

### Induction of apoptosis in human leukemia cells by DATS

**Figure 3:**
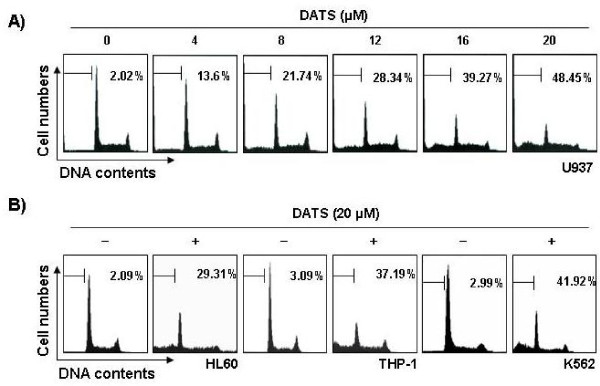
**Increase of sub-G1 cell population by DATS treatment in human leukemia cells.** Cells were treated with various concentrations of DATS for 48 h (A, U937) or incubated with 20 μM of DATS for 48 h (B, THP-1, HL60 and K562).To quantify the degree of apoptosis induced by DATS, cells were evaluated for sub-G1 DNA content, which represents the fractions undergoing apoptotic DNA degradation, using a flow cytometer. Data represent the mean of two independent experiments.

In order to determine whether the decrease in leukemia cell viability by DATS treatment was due to induction of apoptosis, three established criteria were subsequently used for assessment of apoptosis. First, morphological changes of cells were determined using DAPI staining; as shown in Figure 2B and C, treatment with DATS resulted in observation of a significant number of cells with chromatin condensation, loss of nuclear construction, and formation of apoptotic bodies, whereas these features were not observed in control cells. Second, we analyzed DNA fragmentation, which is another hallmark of apoptosis. Following agarose gel electrophoresis of DNAs from cells treated with DATS, a typical ladder pattern of internucleosomal fragmentation was observed. In contrast, DNA fragmentation in control cells was barely detected (Figure 2D). In addition, the degrees of apoptosis in cells treated with DATS were determined using flow-cytometric analysis for detection of hypodiploid cell populations. As shown in Figure 3, addition of DATS to leukemia cells resulted in increased accumulation of cells in the sub-G1 phase in a manner similar to that observed with DATS-induced viability inhibition, formation of apoptotic bodies, and accumulation of extranuclear fragmented DNA. This finding suggests that leukemia cells may undergo apoptosis after exposure to DATS, and there is a good correlation between the extent of apoptosis and inhibition of growth.

### Activation of caspases by DATS in U937 cells

**Figure 4:**
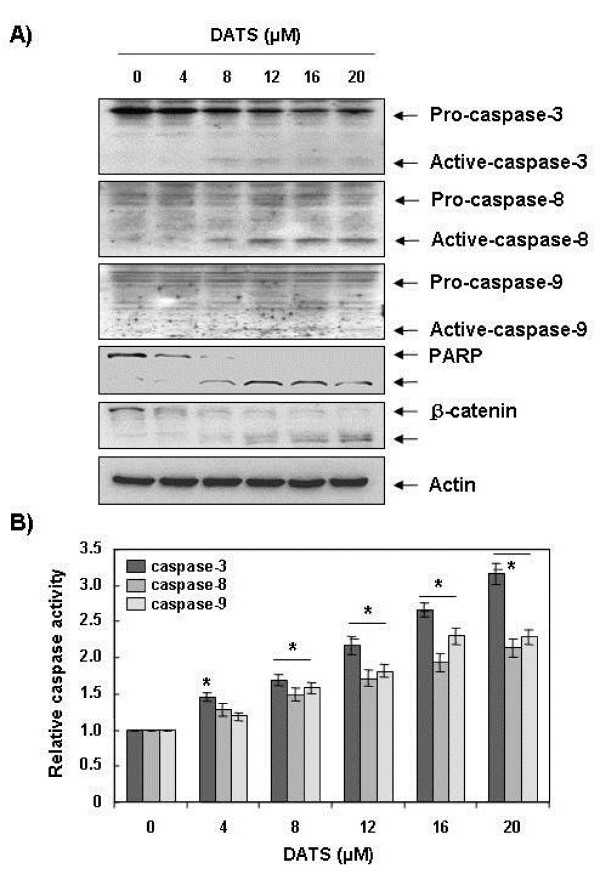
**Activation of caspases and degradation of PARP and β-catenin protein by DATS treatment in U937 cells.** Cells were treated with the indicated concentration of DATS for 48 h. **(A)** Cells were lysed and then equal amounts of cell lysates (30 μg) were separated on SDS-polyacrylamide gels and transferred to nitrocellulose membranes. Membranes were probed with the indicated antibodies. An ECL detection system was used for visualization of proteins. Actin was used as an internal control. **(B)** Cells grown under the same conditions as (A) were collected and lysed. Aliquots were incubated with DEVD-pNA, IETD-pNA, and LEHD-pNA for caspase-3, -8, and −9, individually, at 37 for 1 h. Released fluorescence products were measured. Each point represents the mean ± the SD of representative experiments performed at least three times. A Student’s t-test (*, *p* < 0.05 *vs*. untreated control) was used for analysis of statistical significance of the results.

To reveal the mechanisms underlying the apoptotic effect of DATS on leukemia cells, we determined the expression levels and activities of caspase-3, -8, and −9 using U937 cells. As shown in Figure 4A, immunoblotting results showed that DATS treatment induced a concentration-dependent decrease in levels of procaspase-3, -8, and −9 proteins. For further quantification of the proteolytic activation of caspases, protein in the lysates of cells treated with DATS was normalized and then assayed for *in vitro* activities using fluorogenic substrates. As shown in Figure 4B, treatment with DATS resulted in a significant concentration-dependent increase of the activities of caspase-3, -8, and −9, compared with control cells. In addition, DATS treatment led to progressive proteolytic cleavage of poly(ADP-ribose) polymerase (PARP) and β-catenin, well-known substrate proteins of activated caspase-3, demonstrating an association of DATS-induced apoptosis with caspase activation.

### Effects of DATS on expression of Bcl-2 and IAP family proteins in U937 cells

**Figure 5:**
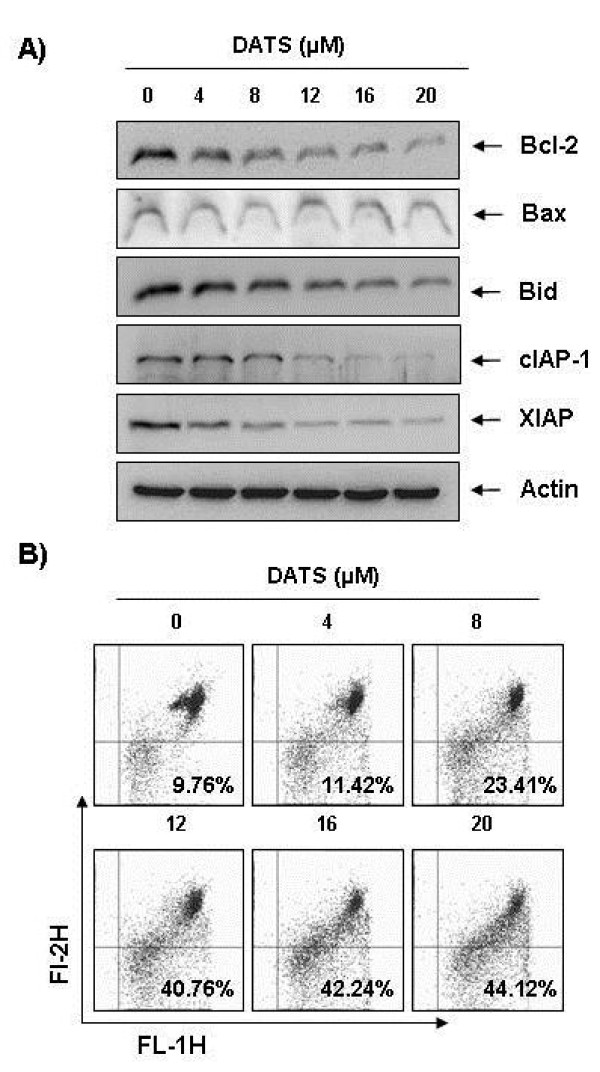
**Effects of DATS on levels of Bcl-2 and IAP family proteins, and MMP values in U937 cells.(A)** Cells were treated with the indicated concentration of DATS for 48 h. Cells were lysed and then equal amounts of cell lysates (30 μg) were separated on SDS-polyacrylamide gels and transferred to nitrocellulose membranes. Membranes were probed with the indicated antibodies and the proteins were visualized using an ECL detection system. Actin was used as an internal control. **(B)** Cells grown under the same conditions as (A) were stained with JC-1 and incubated at 37°C for 20 min. Mean JC-1 fluorescence intensity was detected using a flow cytometer. Data represent the means of two independent experiments.

The role of Bcl-2 and IAP family proteins in DATS-mediated apoptosis was determined by Western blotting for measurement of expression of Bcl-2 and IAP family members. As shown in Figure 5A, the levels of total Bid and Bcl-2 proteins were decreased in response to DATS treatment; however the levels of pro-apoptotic Bax remained unchanged. In addition, the levels of anti-apoptotic XIAP and cIAP-1 were also markedly inhibited by DATS treatment in a concentration-dependent manner.

### Loss of MMP values and increase of ROS generation by DATS in U937 cells

**Figure 6:**
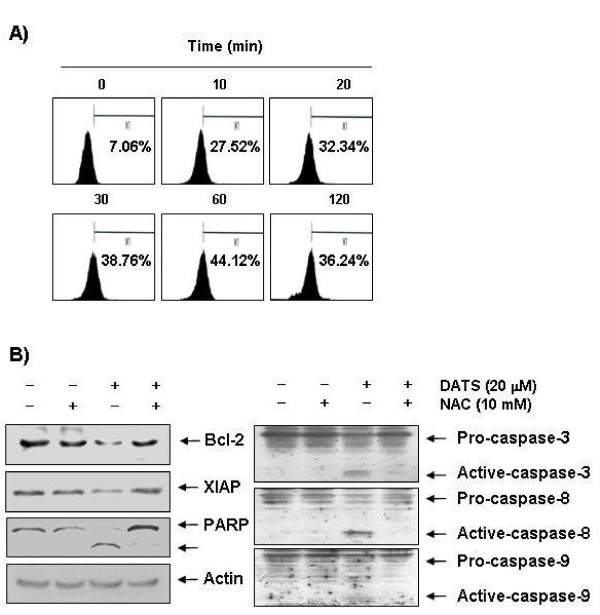
**ROS generation and effects of NAC treatment on modulation of Bcl-2, XIAP, caspases and PARP proteins by DATS in U937 cells.(A)** Cells treated with 20 μM of DATS for the indicated times were incubated with 10 μM of DCFDA at 37°C for 20 min, and ROS generation was measured using a flow cytometer. Results are expressed as the mean of two independent experiments. **(B)** Cells were treated with or without NAC (10 mM) for 1 h before challenge with 20 μM of DATS for 48 h. Cellular proteins were then lysed and separated by SDS-polyacrylamide gels and transferred onto nitrocellulose membranes. Membranes were probed with the indicated antibodies. Proteins were visualized using an ECL detection system. Actin was used as an internal control.

Mitochondria, which play an essential role in apoptosis, are specialized organelles, which contain an outer membrane separated from an inner membrane by an intermembrane space that contains many proapoptotic proteins, including cytochrome *c*. Decrease of MMP causes disruption of the outer mitochondrial membrane, which in turn contributes to release of cytochrome *c.* Because generation and acumination of ROS in cancer cells might be related to mitochondrial dysfunction and cell apoptosis, we attempted to characterize the relationship between ROS production and changes in the MMP. For this study, the effects of DATS on the levels of MMP were monitored via a flow cytometer using the mitochondrial-specific probe, JC-1. As shown in Figure 5B, MMP values showed a concentration-dependent decrease by DATS treatment, indicating that DATS induced mitochondrial membrane hyperpolarization by depolarization. Next, ROS production was measured using a cell-permeant, oxidation-sensitive dye, DCFDA. The results indicated that extending the time of DATS treatment to 0.5 h and 1 h resulted in increased ROS production to greater than 5.5 and 6.2 times that of the control, respectively (Figure 6A).

### DATS-induced apoptosis was associated with generation of ROS in U937 cells

**Figure 7:**
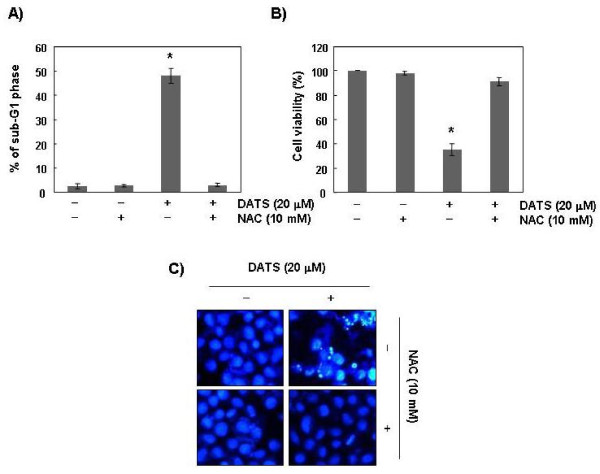
**DATS-induced apoptosis is associated with ROS generation in U937 cells.** Cells were incubated with 20 μM of DATS for 48 h after pretreatment with or without 10 mM of NAC. Cells were evaluated for sub-G1 DNA content using a flow cytometer **(A)** and cell viability was determined using the MTT assay **(B)**. Data are expressed as overall means ± SD from three independent experiments. Statistical significance was determined using the Student’s *t*-test (^*^P < 0.05 vs. vehicle control). **(C)** Following incubation of cells under the same conditions, cells were fixed, stained with DAPI, and the nuclear morphology was then photographed under fluorescence using a blue filter. Magnification, ×400.

In order to show that generation of ROS is a key step in the DATS-induced apoptotic pathway, cells were pretreated with 10 mM of NAC, a commonly used reactive oxygen intermediate scavenger, for 1 h, followed by treatment with DATS for 48 h. Blocking of ROS generation by pretreatment of cells with NAC effectively prevented DATS-induced down-regulation of Bcl-2 and XIAP expression, activation of caspases, and cleavage of PARP (Figure 6B). In addition, NAC had no effect on cell viability and apoptosis induction at a concentration of 10 mM; however, the presence of NAC almost completely suppressed DATS-induced apoptosis, as demonstrated by a near-complete reversal of the percentage of sub-G1 cells that were observed (Figure 7A), which was associated with recovered cell viability (Figure 7B). As expected, blocking of generation of ROS by pretreatment of cells with NAC also prevented DATS-induced chromatin condensation (Figure 7C). Collectively, these findings suggest that an increase in ROS generation is required for occurrence of DATS-induced apoptosis in U937.

## Discussion

Although an increasing amount of data indicate that DATS can suppress the growth of cultured cancer cells by causing cell cycle arrest at G2/M phase and generation of apoptosis [26-29,35-37], little is known about the effects of this compound on the growth of human leukemia cells. In the present study, we demonstrated that DATS-induced anti-proliferative effects in four leukemia cell lines (U937, THP-1, HL60 and K562) were related to induction of apoptosis, as confirmed by measurement of chromatin condensation of nuclei, DNA fragmentation, and induction of sub-G1 phase. Our data also indicated that DATS induced apoptosis of U937 cells through generation of ROS and mitochondrial dysfunction, suggesting that ROS act as upstream signaling molecules for initiation of cell death.

Mounting evidence suggests that damaged mitochondria stimulate increased ROS production, subsequently resulting in activation of the signaling pathways that control cancer cell growth. However, loss of MMP as a result of mitochondrial depolarization in association with apoptosis appears to be more common. The mechanisms by which ROS cause or regulate apoptosis typically include caspase activation and modulation of Bcl-2 family protein expression [38,39]. Caspases, a family of cystein-containing aspartate-specific proteases, are known to play key roles during apoptosis and to lead to initiation and execution of apoptosis. Activation of initiator caspases, such as caspase-8 and −9, resulted in downstream activation of effector caspases, such as caspase-3 and −7 [40,41]. The decrease in MMP causes disruption of the outer mitochondrial membrane and contributes to release of cytochrome *c*. Release of cytochrome *c* has been reported to contribute to activation of caspase-9, which in turn causes activation of caspase-3. In particular, activation of capase-3 is responsible for proteolytic degradation of many key proteins, including PARP and β-catenin, finally leading to apoptosis [42,43]. Modulation of anti- and pro-apoptotic proteins of the Bcl-2 family also controls mitochondrial function. In mammals, members of the Bcl-2 family can be divided into two subfamilies; the anti-apoptotic protein family, including Bcl-2, and the pro-apoptotic protein family, including Bax. Balance between anti-apoptotic and pro-apoptotic members also determines the fate of the cell through mitochondrial dysfunction [44,45]. In addition, activation of caspase-8 by apoptotic stimuli converts Bid to truncated Bid (tBid), leading to conformational changes in Bax, mitochondrial depolarization, and release of cytochrome *c* from mitochondria. This leads finally to activation of caspase-3 and induction of apoptosis via a complex of apoptotic protease activating factor-1 (Apaf-1), procaspase-9, and cytochrome *c* after translocation of tBid to the mitochondria [41,44-46]. Furthermore, members of the IAP family, which includes XIAP, cIAP-1, and cIAP-2, have been reported to exert their anti-apoptotic effects due to their function as direct inhibitors of activated caspases. Therefore, down-regulation of IAPs relieves the triggering block of proapoptotic signaling and execution of caspases, thus activating cell death [47,48].

In this study, our data indicated an association of DATS-induced apoptosis of U937 cells with increased generation of ROS and enzymatic activity of both the extrinsic and intrinsic caspase cascades, including caspase-8 and −9. Although the truncated form of Bid fwas not detected, the levels of intact Bid proteins were gradually down-regulated by DATS in a concentration-dependent manner. DATS also caused a significant reduction in MMP values, which was connected with activation of caspase-3 and concomitant degradation of PARP and β-catenin. In addition, down-regulation of anti-apoptotic Bcl-2 and IAP family proteins, including Bcl-2, XIAP, and cIAP-1, was observed in U937 cells exposed to DATS, as compared with control cells. Thus, the results indicated that caspase-8 activation by DATS appeared to trigger mitochondrial apoptotic events by inducing conformational changes in apoptotic proteins.

ROS-mediated caspase activation and mitochondrial dysfunction have been suggested as critical for DATS-induced apoptosis in several cancer cell lines [26-29]; however, the current role of mitochondrial functional changes associated with ROS generation in the response of human leukemia cells to DATS has not yet been explored. Therefore, we next investigated the question of whether these observations and apoptosis by DATS in U937 cells were associated with generation of ROS. The results revealed that activation of caspase-9 and −3, degradation of PARP, and inhibition of Bcl-2 in DATS-treated cells were ROS-dependent and that co-culture with NAC, a commonly used ROS scavenger, effectively blocked DATS-induced apoptosis in U937 cells. Findings from the present study also indicated that activation of caspase-8 in DATS-treated cells is ROS-dependent, which suggests that ROS may act upstream of caspase-8 activation in U937 cells. Therefore, it is reasonable to assume that the initial signal for activation of caspase-8 after treatment with DATS is also derived from ROS. In addition, blocking of ROS generation prevented DATS-induced down-regulation of XIAP and cIAP-1. Because IAP family proteins are also substrates of caspase-3 [49,50], the observed decrease in XIAP and cIAP-1 expression may be due to caspase-3-mediated processing following DATS treatment. Thus, our data indicate that ROS production and mitochondrial dysfunction are possible contributing factors to DATS toxicity.

## Conclusions

In summary, the present study demonstrated that human leukemia cells undergo apoptosis in response to treatment with DATS, and that this occurs through a mitochondria-mediated pathway that requires ROS generation upstream for disruption of the MMP, which leads to subsequent activation of caspases. Although in this paper we only performed a rough assessment for determination of whether ROS was involved in the apoptosis induced by DATS, our data emphasize the key role of ROS in apoptosis induced by DATS in U937 cells, and indicate that a positive correlation exists between ROS and mitochondrial events leading to apoptosis, and may aid in understanding of the mechanisms for the anti-cancer activity of DATS.

## Competing interests

The authors declare no potential conflict of interests.

## Authors’ contributions

HSP carried out major experiments in the study and helped to draft the manuscript with YHC. YHC contributed to the experimental design, data interpretation, editing, and submission of this manuscript. All authors read and approved the final manuscript.
